# Class 2 CRISPR/Cas: an expanding biotechnology toolbox for and beyond genome editing

**DOI:** 10.1186/s13578-018-0255-x

**Published:** 2018-11-12

**Authors:** Yuyi Tang, Yan Fu

**Affiliations:** 1 MicroAnaly (Shanghai) Gene Technologies Co., Ltd, Shanghai, China; 2 Anhui MicroAnaly Gene Technologies Co., Ltd, Chaohu, Anhui China; 3 National Gene Research Center, Chaohu, Anhui China

**Keywords:** Genome editing, Transcriptional repression, Diagnostic detection, CRISPR, Cas9, Cas12a, Cas13

## Abstract

Artificial nuclease-dependent DNA cleavage systems (zinc-finger nuclease, ZFN; transcription activator like effectors, TALENs) and exogenous nucleic acid defense systems (CRISPR/Cas) have been used in the new era for genome modification. The most widely used toolbox for genome editing, modulation and detection contains Types II, V and VI of CRISPR/Cas Class 2 systems, categorized and characterized by Cas9, Cas12a and Cas13 respectively. In this review, we (1) elaborate on the definition, classification, structures of CRISPR/Cas Class 2 systems; (2) advance our understanding of new molecular mechanisms and recent progress in their applications, especially beyond genome-editing applications; (3) provide the insights on the specificity, efficiency and versatility of each tool; (4) elaborate the enhancement on specificity and efficiency of the CRISPR/Cas toolbox. The expanding and concerted usage of the CRISPR/Cas tools is making them more powerful in genome editing and other biotechnology applications.

## Background

### Introduction of CRISPR/Cas

Clustered regularly interspaced short palindromic repeats (CRISPRs), formerly known as short regularly spaced repeats (SRSRs) [1], are a family of repetitive DNA sequences. They contain 21–37 bp direct repeats (DRs) interspaced by similarly sized non-repetitive and variable sequences or spacers [2, 3]. A CRISPR locus (i.e. a DR-spacer array) is flanked by a leader sequence (LS) on its 5′ end. The DRs and LS have been found conserved within the same species, while varied across species. Within the same species, the DRs of different strains had evolved via the interstitial deletion of motifs, but still could be traced back to a common ancestor [4–6]. CRISPR-associated (*Cas*) genes adjacent to a CRISPR locus encode a series of Cas proteins [3] that have functional relationships with each other. CRISPRs and CRISPR/Cas systems are found present in almost all archaea and ~ 40% of bacteria [7], but absent from eukaryotes or viruses [3]. The CRISPR/Cas systems have a “memory/immune function” so that the bacteria host can “store” the information of attacking foreign nucleotides and then specifically identify and cleave the “invaders” when it is threatened again. In other words, these prokaryotes obtain acquired immunity from the adaptive CRISPR/Cas systems against exogenous invasion (e.g., bacteriophages and plasmids) via integrating “ID” sequences of foreign nucleic acid into new motifs.

**Fig. 1:**
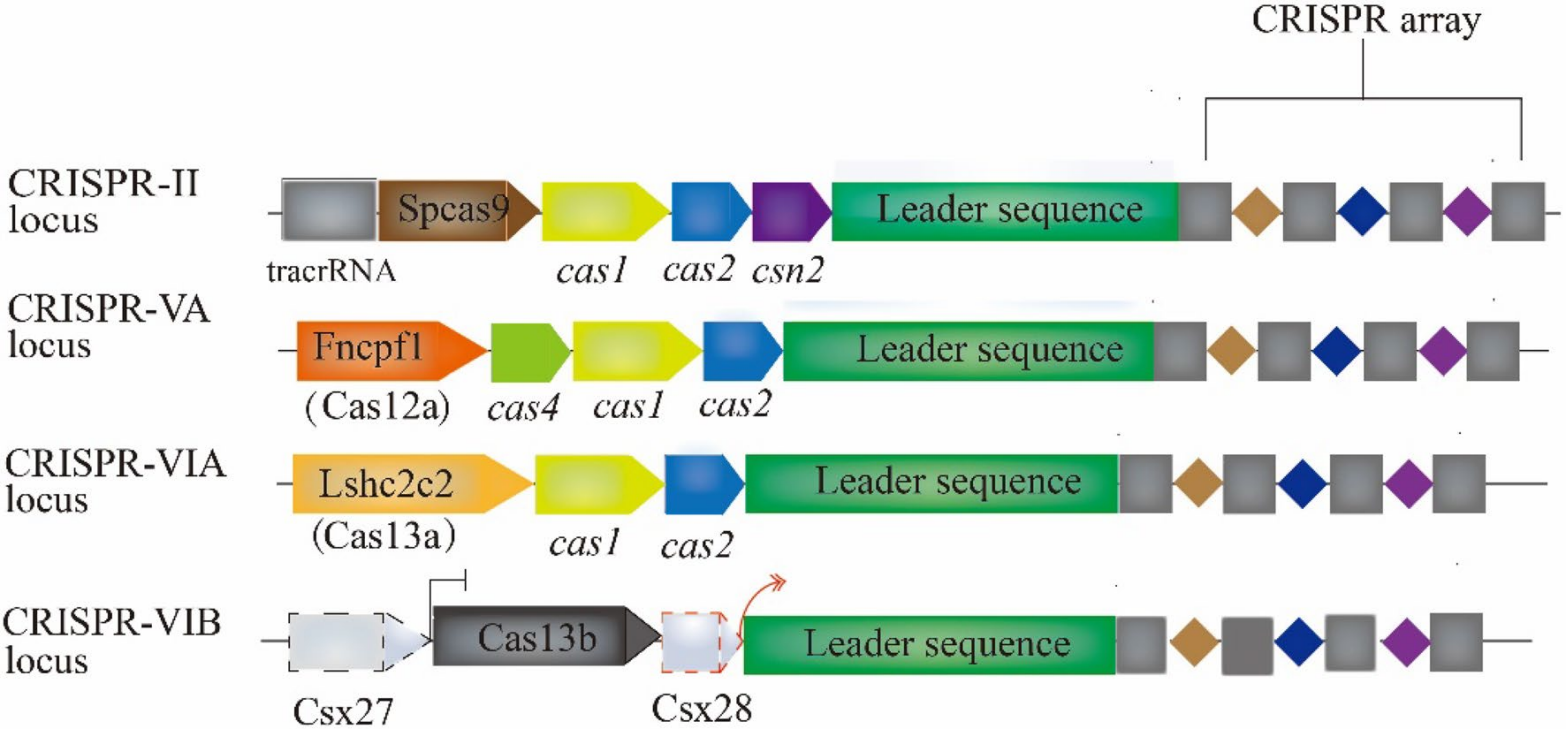
The genomic organization of generalized CRISPR/Cas loci in Class 1, Type II and Class 2 systems, illustrated according to Ref. [8, 120, 121]. The organization contains CRISPR-Cas loci and domain architectures of the effector proteins. Compared to Class 2 systems, Type II Cas9 systems need tracrRNA. Except for Type VI-B, Type II, VA and VIA all have their own CRISPR/Cas genes. For subtype VI-B, the activity of the effector protein Cas13b could be repressed by Csx27 protein and enhanced by Csx28

CRISPR/Cas systems so far have been be grouped into two classes, six types and over thirty subtypes [8], based on the constitution of effector protein (the class level) and the presence/absence of signature genes, protein sequence conservation, and organization of the respective genomic loci (the types and subtypes). Class 2 is characterized by only one effector protein whereas Class 1 contains multi-subunit Cas protein complexes. It appears that the Class 2 systems have more potentials in applications of gene editing and genetic screening, demonstrated by numerous studies and applications using Cas9 (Csn1), Cas12a (Cpf1), Cas13a (C2c2) and Cas13b (C2c6) systems. Several conserved genes (e.g. *cas1* and *cas2*) in the vicinity of CRISPRs involved in DNA recombination and repair. The *cas3* gene has motif characteristics of helicases of the superfamily 2, and the *cas4* gene has motifs of the RecB family of exonucleases, suggesting that these genes are involved in DNA metabolism or gene expression [3]. Four major programmable systems are composed of different backbones (Fig. 1) and elucidated in the sections below.

## CRISPR/Cas9

**Fig. 2:**
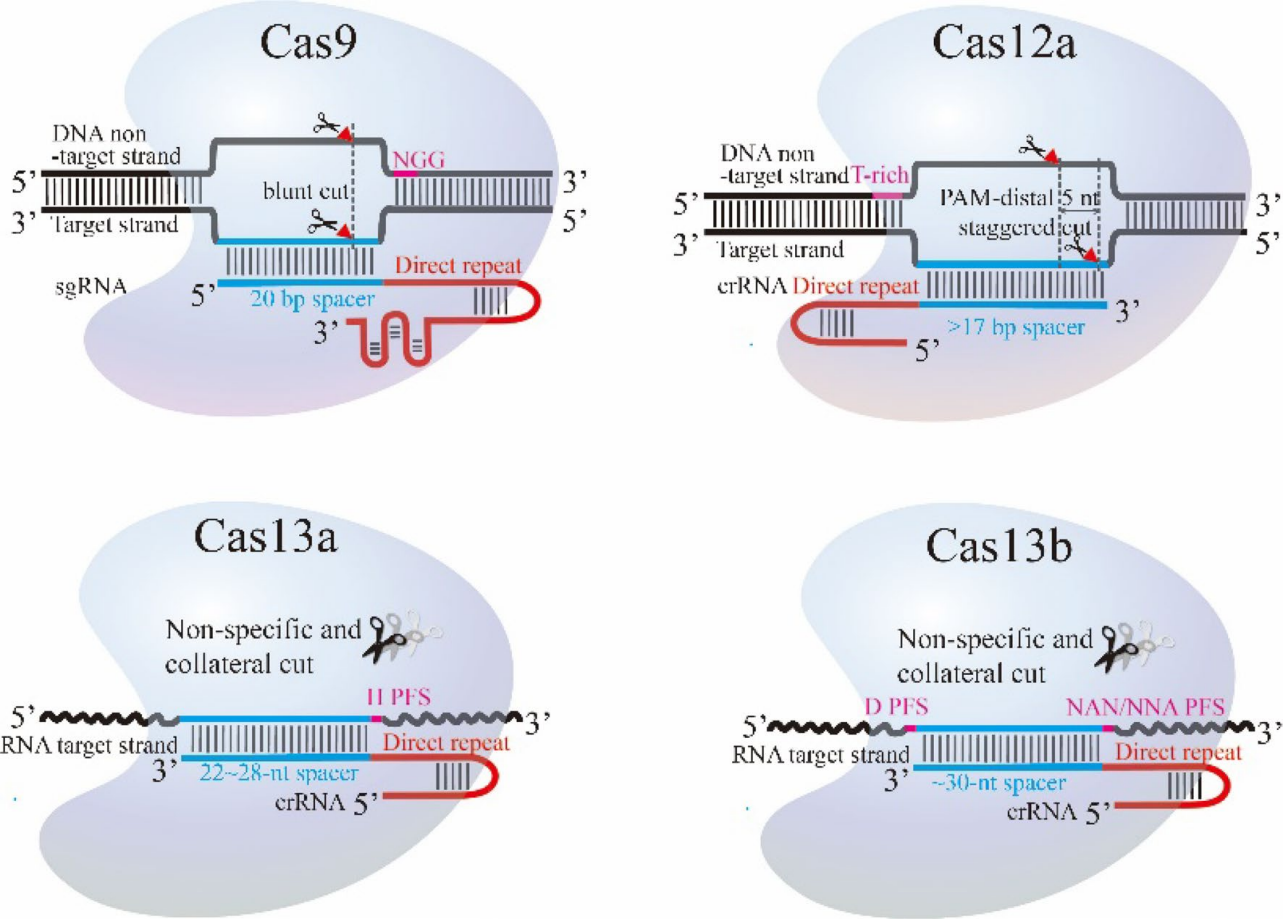
Diagram of the CRISPR/Cas9, CRISPR/Cas12a and CRISPR/Cas13 genome editing protocol. DRs (red) and spacer (blue) constitute the sequence of sgRNA (in Cas9 systems) or crRNA (in Cas12a and Cas13 systems). Optimal PAMs or PFSs highlighted in pink are critical for target recognition of these corresponding systems. In Cas9 staggered cleavage pattern, HNH cleaves TS to generate blunted end while RuvC cleaves NTS to generate non-blunted ends with 5′ 1- to 3-nt overhangs. The cleavage of Cas12a and Cas13 systems is in a staggered and collateral manner, respectively

**Fig. 3:**
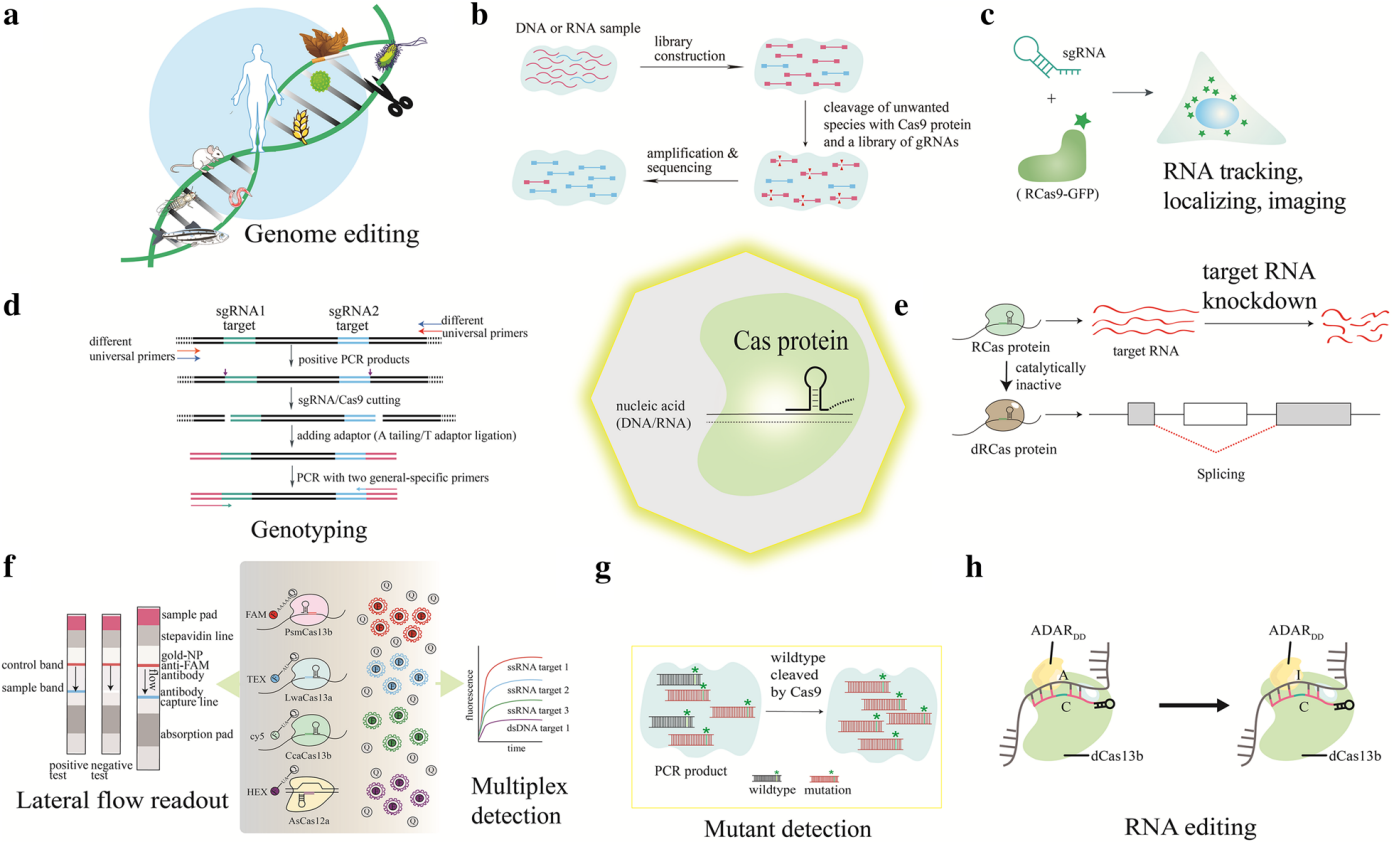
The new genome engineering and other biotechnology applications of CRISPR/Cas systems. **a** Robust genome editing with CRISPR/Cas, especially Cas9 in microbe, plant, animal, human cells. **b** The target sequence enrichment or normalization with Cas9 cleavage in NGS libraries, modified from Ref. [56]. **c** Usage of sgRNA/RCas effectors (RCas9)-GFP in RNA tracking, localizing and imaging in cells, modified from Ref. [133]. **d** Combination of PCR and sgRNA/Cas9 cutting followed by A tailing and T adaptor ligation for genotyping, modified from Ref. [58]. **e** RNA knockdown with RCas effectors (Cas13d) and splicing with catalytically inactivated dCas13d, illustrated according to Ref. [132]. **f** Multiplex detection achieved by the combination of Cas12a, Cas13a and Cas13b with different cutting behaviours, and naked-eye readout of lateral flow detection, modified from Ref. [115]. **g** More accurate mutant detection with PCR amplification after enrichment by Cas9 cleavage, illustrated according to Ref. [59]. **h** crRNA/dCas13b fused with ADARDD (an adenosine deaminase acting on RNA) for RNA editing, modified from Ref. [120]

CRISPR/Cas9 system has been the first and most widely adopted for genetic engineering. It contains Cas9 protein, crRNA and tracrRNA. Cas9 protein is analogous to a bilobed jaw in shape which contains one recognition (REC) lobe and one nuclease (NUC) lobe [9]. The REC lobe, composed of a long bridge helix, a REC1 domain and a REC2 domain, is a DNA-targeting recognition site. The NUC lobe has RuvC, HNH and PI domains [9–11]. The HNH nuclease is inserted into RuvC domain and initiates the cleavage of the DNA strand complementary to the guide RNA, while RuvC cleaves the other strand (i.e. the system targets and cleaves dsDNA) [12]. The PI domain can recognize the protospacer adjacent motif (PAM) sequence on the noncomplementary strand to form an R loop. PI domain is critical for PAM specificity. Pre-crRNA and tracrRNA are transcribed from CRISPR and DRs, respectively. Cas9 protein is involved in the tracrRNA-mediated processing of pre-crRNA into mature crRNA. The 5′-terminal of crRNA is complementary to the targeting site, and its 3′-terminal can form complexes with Cas9 and tracrRNA. The Cas9-tracrRNA-crRNA complex plays an important role in recognition and binding of Cas9 on target sites and specific cleavage. A PAM adjacent (either up- or downstream) to the target site required for interference by the systems is varied in subtypes and typically 3′GC-rich (Fig. 2) [8, 13]. More detailed representation about the complexes of the Class 2 effector proteins with the target and guide RNA is available in Koonin’s artwork (https://www.sciencedirect.com/science/article/pii/S1369527417300231, Fig. 3a) [8].

### Applications of CRISPR/Cas9 on genome-editing

To simplify the protocol of CRISPR/Cas9 genome-editing, a single guide RNA (sgRNA) is designed to function as the crRNA-tracrRNA complex. In this simplified system, Cas9 can recognize sgRNA and then be guided to bind the target sites. Doudna and Charpentier group demonstrated the ability of CRISPR/Cas9 system to cleave both DNA strands in vitro and modify genes in living cells, and proposed the potentials of CRISPR in genome editing [11]. Previous studies reported that CRISPR/Cas9 system cut both DNA strands, resulting in blunt ends at a position three base pairs upstream of the PAM sequence [10, 11, 14]. This cutting pattern was once thought to be a weakness of Cas9 system. However, a recent finding of staggered Cas9 cleavage demonstrated that HNH cleaves TS to generate blunted while RuvC cleaves NTS non-blunted ends with 5′ 1- to 3-nt overhangs in vivo and in vitro (Fig. 2) [15]. After double strand break (DSB) at the target site is induced by Cas9/sgRNA cleaving, cells initiate DNA repair via either homology-directed repair (HDR) or non-homologous end joining (NHEJ). HDR happens only when the homologous sequences exist and frequently results in target-site correction or insertion of foreign DNA through homologous recombination between donate DNA and host genomic DNA. NHEJ takes place without the need for a homologous template and often cause loss of gene function in the cleaved region [16, 17]. Both loss-of-function (e.g. Tay-Sachs disease) and gain-of-function mutations (e.g. Huntington disease) could be corrected by HDR [18], on which Cas9-mediated therapeutic genome editing is based. As a genome editing tool (Fig. 3a), CRISPR/Cas9 system has been successfully applied in virus [19–21], bacteria [22–25], yeast [26–32], plant [33–37], nematode [38], *Drosophila* [39], zebrafish [40], frog [41], chicken [42], mice [43], sheep [44], monkey [45] and human [46], etc.

Undoubtedly, Cas9 systems hold a potential to solve some great challenges in modern medicine. Taking the HIV study by the Hu and Khalili’s groups as an example, they used Cas9/sgRNA to target the highly conserved long terminal repeats (LTRs) of the HIV-1 proviral DNA, inactivated their expression and replication in the microglial, promonocytic, and latently infected T cells in an in vitro study [47, 48]. Following that, the CRISPR/Cas9-based machinery that eliminates 5′ and 3′ LTRs of HIV-1 genomes was delivered in the ex vivo culture of latently infected human CD4+ T-cells and achieved the suppression of HIV-1 replication and reduced viral load [49]. Further success of eradicating the key segments of the HIV DNA was achieved in transgenic mice and rats with HIV-1 by employing a shorter version of the Cas9 endonuclease and a multiplex of sgRNAs to target the viral DNA sequences within the 5′-LTR and the *Gag* gene [50]. In addition, Duchenne muscular dystrophy (DMD) and cardiac diseases were challenged by using CRISPR/Cas9 system to remove specific gene regions from the mouse host genome [51, 52]. The mutants of Cas9 with two inactivated endonuclease domains (dCas9, dead Cas9) retain the ability of target-binding. Epigenetic modifications have been achieved by using dCas9 fusions with histone modifiers [53] and proteins (MQ1) [54] for selective DNA methylation or demethylation (TET1CD) [55], which can be applied in epigenetics-based cancer research as well as AIDS, DMD, and cardiac diseases.

### Applications of CRISPR/Cas9 beyond genome-editing

Beyond the genome engineering applications abovementioned, it’s worth noting of the revolutionary applications of Cas9 system into the next-generation sequencing (NGS) fields (Fig. 3b). Using Cas9 system, more than 99% of unwanted sequences with high abundance in sequencing libraries could be removed [56]. In contrast to other depeletion/normalization methods, it depletes abundant sequences after cDNA amplification so that it has no limitation by input sample amount. This method finally addressed the current limitation that low pathogen load presents as a minuscule fraction of the total. Another application of CRISPR combined with NGS technology is short tandem repeat (STR)-Seq. CRISPR/Cas9-mediated enrichment of the DNA fragments that span the targeted microsatellite loci was achieved and over 2000 STRs were sequenced and typed in parallel by NGS. STR-Seq greatly facilitated the studies on STR-related diseases and genetic identification in forensics [57]. Another interesting application of Cas9 system is molecular diagnostics. Cas9 system was recently used in HPV16/18 DNA detection and genotyping (Fig. 3d). The protocol includes PCR amplification with a universal primer pair, Cas9 cutting, A tailing and T adaptor ligation. After determining if target virus DNA exists, the amplification was performed with a pair of HPV16- or HPV18-specific primers to distinguish the subtypes of HPV [58]. Moreover, mutation signal could be similarly raised after the cleavage of wildtype fragments (Fig. 3g). 0.1% of the *EGFR* E746-A750 deletion could be detected via combining Cas9 treatment and blocker PCR [59].

The dCas9 systems can also be used for the research beyond genome-editing. For example, live-cell imaging of genomic elements is necessary to study their physical organization and interactions with other elements. It has been a major challenge to visualize arbitrary, endogenous DNA sequences in living cells until dCas9-GFP fusions were adapted [60]. Ma et al. [61] established a system for imaging multiple genomic loci in living cells based on dCas9 combined with sgRNA scaffolds that bind fluorescent proteins. As a CRISPR-activation (CRISPRa) or CRISPR-inhibition (CRISPRi) tool, dCas9 systems could be used for: (1) transcriptional repression via blocking the transcription of the specific targeted gene loci bound by dCas9/sgRNA, or fusing KRAB at the C-terminal of dCas9; (2) transcriptional activation via fusing p65AD at the C-terminal of dCas9.

CRISPR/Cas9 had been widely exploited in DNA only till researchers successfully tweaked it to target RNA. Cas9 protein from *Francisella novicida* (FnCas9) was found to be guided to a specific mRNA by the tracrRNA and a small CRISPR/Cas-associated RNA (scaRNA), leading to the repression of the transcript [62]. Another type of RNA-targeting Cas protein, Cas13, has two higher eukaryotes and prokaryotes nucleotide-binding (HEPN) domains that are responsible for RNA targeting and cleavage while FnCas9 has no HEPN domain. The RNA-binding arginine-rich motif might be necessary for FnCas9 to interact with RNA. There exist other two models that endogenous RNases are recruited to the target by FnCas9, and that FnCas9 has an additional domain with endonucleolytic activity [62]. Either FnCas9 or Cas13 requires a guiding RNA for RNA targeting [63]. The speculation that FnCas9 may facilitate programmable RNA targeting was later confirmed in eukaryotic cells [63, 64] including plants [65]. However, the application of FnCas9 for targeting RNA has been sluggish because its nuclease function could be inhibited much more frequently in living human cells, compared with SpCas9 [66]. A more flexible method has been established to enable commonly used Cas9 systems (i.e. SpCas9) to target and cleave RNA: the RNA-targeting Cas9 (RCas9) utilizes not only the inherent endonucleolytic activity of Cas9 to eliminate gene expression by cleaving particular transcripts, but also a PAM-presenting oligonucleotides (PAMmer) that can partially bind to the target RNA [67]. The PAMmer enables RNA-targeting rather than DNA, different from the fashion that the PAM use to ensure only foreign DNA recognition. However, RNA–DNA heteroduplexes formed between the mRNA and PAMmer could be cleaved by cellular RNase H. Under such circumstances, the PAMmer was chemically modified using a 2′-OMe modification method to eliminate RNase-H-mediated cleavage. The dCas9 systems are frequently harnessed in sequence-specific and programmable control at transcriptional level [11, 68]. More recently, the versatile RNA-targeting Cas9 tool (Fig. 3c) has been applied for localizing, imaging and tracking [69] and abundance measurement in living cells [70].

### Off-target of Cas9: the issue of specificity

The issue of targeting specificity must be taken into account for all nucleotide recognition tools including CRISPR/Cas9. The reason for its high off-target rates is that the length of the target sequence recognized by sgRNA is only 20 bp and Cas9 protein is not sensitive to the mismatch(es) between the target site and the 5′-terminal of sgRNA. Several strategies have been taken to solve this issue: (1) the paired D10A Cas9 nickases with offset sgRNAs [17, 18] enhanced genome editing specificity [71, 72]; (2) sgRNA truncation at 5′-terminal to lower the binding energy to the recognition site with mismatch(es) when introducing a long sgRNA with high binding energy [73]; (3) a FokI nuclease domain fused to a pair of catalytically-inactive Cas9 nucleases to achieve much higher specificity of target sequence recognition [74]; (4) neutralization of the positive charges in the non-target strand groove (nt-groove) of Cas9 to decrease off-target indel formation while preserve on-target activity [75]; (5) an evolved SpCas9 variant with the broadest PAM compatibility, xCas9, was rapidly obtained by using phage-assisted continuous evolution (PACE). Compared with the wild type Spcas9, xCas9-3.7 and -3.6 offered greatly reduced off-target activity, exhibiting much higher specificity despite their broader PAM compatibility [76].

It was observed that the inefficient delivery of CRISPR reagent could exacerbate off-target effect in cultured cells or local tissues in vivo. To overcome this obstacle, lentiCRISPR v2 plasmid was re-engineered to reduce off-target effect by making a self-restricted CRISPR system with a second gRNA co-expression cassette inserted [77].

## CRISPR/Cas12a

Class 2 Type V-A Cas12a system (formerly known as Cpf1), is composed of an ordered *cas12a*-*cas4*-*cas1*-*cas2*-CRISPR array. Similar to Cas9 in size and shape, Cas12a protein has two RuvC nuclease domains that could even be superimposed. The Cas12a protein contains a distinct nuclease domain inserted into a similar but not identical position within the RuvC-like domain instead of the HNH domain. The abovementioned Koonin’s artwork also illustrated that tracrRNA is necessary for all Type II, but not for a few of Type V systems like Cas12a [8]. Cas12a has been studied on how it mediates robust DNA interference in a different fashion from Cas9 [78]. In silico prediction shows that FnCas12a crRNA from *Francisella novicida* contains 19 nt DR fragments, a 23–25 nt spacer sequence, and a single stem-loop [78] with a pseudoknot structure [79]. Cas12a alone is sufficient for crRNA maturation in vitro [78, 80]. Cas12a can cleave both RNA and DNA. Before DNA cleavage happens, RNA cleavage is conducted with the presence of the crRNA produced from the first reaction [80]. The PAMs for Cas12a and Cas9 are at the opposite end of their own protospacer (Fig. 2). A short T-dependent PAM, such as 5′-YTN-3′, 5′-TTN-3′or 5′-TTTN-3′ (the T in the middle is more important than the first T), is required for the single-RNA guided Cas12a system for efficient target DNA cleavage. The PAM recognition mechanism in Cas12a system was revealed as a combination of base and shape readout [79]. The target and non-target DNA strands (TS and NTS) are cleaved by a single RuvC domain [81], not by both RuvC and the nuclease domain [79]. The cleavage site of FnCas12a is after the 18th base on the NTS and at, or after the 23rd base on the TS, away from the PAM. The staggered DNA DSB with a 4 or 5-nt 5′ overhang generated from cleavage can further stimulate the NHEJ repair [78, 82]. The DR of mature crRNA has been found to be at least 16 nt in length and reach maximum cleavage efficiency in length of 17–18 nt.

### Applications of CRISPR/Cas12a on genome-editing

Based on the enzymatic features of Cas12a, its function was initially tested in *Escherichia coli* and later explored for robust genome-editing applications in human cultured cells [78]. Subsequent researchers quickly adapted Cas12a system in rice and tobacco [83–87], cyanobacteria [88], mice [89–92], *Saccharomyces cerevisiae* [93], *Corynebacterium glutamicum* [94], and *Bombyx mori* [95]. Although Cas9 system is the mostly used tool in CRISPR, Cas12a has shown several marked advantages over it as follows:

i. At least one G must be present if exploiting Cas9 family whereas the T-dependent PAMs of Cas12a-family proteins expanded the targeting range of genome editing, especially in targeting the organisms with AT-rich genomes, such as *Plasmodium falciparum* [96], malaria parasite and hyperthermophiles, etc., or A/T-rich regions such as scaffold/matrix attachment regions [97].
ii. It is indicated that Cas9 possesses cytotoxicity upon genome editing of some organisms such as *Corynebacterium glutamicum* (*C. glutamicum*) [94] and cyanobacteria [88]. Given that the inherent toxicity of Cas9 to cells prevents it from being used for genome editing in these organisms, Cas12a exhibits no obvious toxicity and facilitates the gene editing in various species like *C. glutamicum* with high efficiency, thus offers an excellent alternative [88].
iii. Compared with multiplex genome editing of Cas9 that requires relatively large constructs or simultaneous delivery of multiple constructs, Cas12a with a single customized crRNA array can be used to simultaneously edit up to four genes in mammalian cells and three in the mouse brain [98]. Only one Pol III promoter is required for Cas12a nuclease to drive several small crRNAs (39 nt per crRNA) in multiplex gene editing of rice [85] and *Saccharomyces cerevisiae* [93]. A recent publication reported that the editing efficiency mediated by Pol II (cytomegalovirus; CMV) promoter-drived crRNAs is higher than the one by a Pol III promoter-drived crRNAs [99]. From the point of cost-efficiency, the Cas12a system with a smaller RNA component (42–44 nt) costs ~ 60% less than Cas9 systems with over 100 nt sgRNA synthesized [78], without compromising any efficiency. Moreover, multiplex gene regulation could be facilitated by employing ddCas12a in the same way.
iv. Because Cas12a generates staggered ends distant from the critical seed region, NHEJ will not disrupt the target site and thus Cas12a can recut the same site until the desired HDR recombination event take place. It is demonstrated that this property can increase the efficiency of the system in plants [88, 100] and is later used for editing genetic mutations in human and mice genomes [92].
v. Cas12a induced relatively larger mutagenic indels than the majority of Cas9-induced mutations [101], which may benefit the functional analyses of noncoding (e.g., miRNA) genes, regulatory DNA elements, and large desired regions.
vi. Cas12a generates cleavage products with 2′,3′-cyclic phosphate ends, which could help activate the CRISPR Type-III effector nuclease Csm6 cleavage and then amplify the signal for multiplex detection.

### Controversy about CRISPR/Cas12a

However, there is some controversy about the Cas12a system. It has been demonstrated that the relaxed PAM does not lead to increased off-target cutting, at least in human cells. The results showed that Cas12a was sensitive to single mismatch at positions 1–18 in the 5′-PAM proximal region where double mismatches could even induce a nearly complete loss of Cas12a activity but tolerated single or double mismatches in the 3′-PAM-distal region [82, 102]. In the CRISPR/Cas12a system, indel frequencies at off-target sites can largely be minimized by truncating four to six bases of crRNAs at the 3′ end without sacrificing their on-target counterparts. But most off-target sites harbor mismatches at the PAM-distal 3′ end, which limited the application of CRISPR/Cas12a system [82, 102]. Challenges remain in the lower on-target cleavage efficiency for Cas12a system than the better studied Cas9 [82]. With a more optimistic view, Fonfara et al. [80], who proposed that mismatches around the target site might reduce cleavage activity, thought that Cas12a is more sensitive to mismatches within the target site compared to Cas9. Regarding indels frequency, Begemann et al. [100] reported that the frequency of targeted insertion by HDR in plants for the Cas12a nucleases, up to 8%, was higher than most other genome editing nucleases including Cas9, indicating its enzymatic effectiveness. The paradox is that, Cas12a preferentially creates deletions as opposed to insertions [82, 83]. Surprisingly, Xu et al. [86] found that transforming with pre-crRNA could enable higher mutation efficiency than the use of mature crRNA in rice, suggesting that pre-crRNA may be more critical than mature crRNA for targeting plant genes. Port and Bullock [103] successfully enhanced genome editing of Cas12a in vivo by flanking active sgRNAs with tRNAs. How tRNAs can increase the activity of Cas12a sgRNAs remains unknown.

## CRISPR/Cas13

### CRISPR/Cas13a

While most CRISPR/Cas systems target dsDNA, Types VI and III are specialized or pluralistic for RNA interference [104]. Cas13 (including Cas13a and Cas13b), the archetypal protein of Type VI, lacks a DNase domain that Cas12a/Cas12b/Cas9 has. Instead, two HEPN domains are located on the external surface of Cas13 (Fig. 2) [105, 106].

As a dual ribonuclease, Cas13a can cleave pre-crRNA to generate crRNA maturation [107], and the helical-1 domain in LshCas13a and the HEPN2 domain in LbuCas13a are likely involved in pre-crRNA processing [108, 109]. Although an in silico approach first predicted Cas13a loci includes the adaptation-related genes *cas1* and *cas2*, Feng Zhang’s group subsequently showed that the majority of Cas13a loci consist only of the *Cas13a* gene and a CRISPR array [110]. It is worth noting that the apparent incomplete loci could still encode defective CRISPR-Cas systems and function with the adaptation module encoded elsewhere in the genome, as observed for some Type III systems [111].

Taking a big step for future RNA research, Abudayyeh and colleagues leveraged Cas13a (LshCas13a) as a novel programmable RNA-targeting endoribonuclease [112]. Their results showed that LshCas13a cleaves ssRNA exclusively, upon recognizing target sequences of 22–28-nt complementary to the crRNA spacer that must contain one at least 24-nt-long stem-loop structure. The target sequence must be flanked by a mononucleotide protospacer-flanking site (PFS) at the 3′-end, having bias to A, U or C (Fig. 2). Notably, LshCas13a and crRNA bind together to generate a duplex that then binds the target and preferentially cleaves exposed regions (e.g., loop regions) of ssRNA at uridine (or adenosine for LbaCas13a, EreCas13a and CamCas13a) without tracrRNA [113]. In this system, a single mismatch across the spacer can be tolerated, but two mismatches distributed in the central region of the spacer can dramatically reduce the target RNA cleavage efficiency. Additionally, LshCas13a-crRNA duplex could cleave other ssRNA in a non-specific manner once activated by target ssRNA, which is referred to “collateral effect”. Liu et al. [108] found that target RNA binding induced conformational changes on the Helical-2, HEPN1, and Linker domains in Cas13a. The conformational changes generated a guide-target RNA duplex binding channel, and then activated Cas13a to cleave target and collateral RNAs. East-Seletsky et al. [113] suggested that pre-crRNA processing is not necessary for targeting but enhances cleavage activity by liberating crRNAs from the CRISPR array. Whether other type VI CRISPR-Cas enzymes have the same activation process of collateral cleavage and the same role of pre-crRNA processing in cleavage activity as Cas13a, is needed to be studied in the future.

### Applications of CRISPR/Cas13a beyond genome-editing

Notably, ingenious use of the seeming shortcoming may broaden the versatility and feasibility of CRISPR tools. Given that ultra-high sensitivity is a compelling need for many diagnostic applications, Zhang’s research group leveraged the collateral effect and isothermal amplification (recombinase polymerase amplification, RPA) to open up a new avenue for precise CRISPR-based diagnostics (CRISPRdx) [114]. The CRISPRdx technology, also called “specific high sensitivity enzymatic reporter unlocking (SHERLOCK)”, specifically detected ssRNA/ssDNA at attomolar level. They substituted LwCas13a for LshCas13a to obtain robust signals. Firstly, the signals of RNA samples were transcribed and amplified with RT-RPA. Secondly, Cas13a-crRNA complex targeted and cleaved the target sequence before the reporter RNA was cleaved collaterally and then released the signals. With regard to DNA targets, DNA template could be amplified with RPA first. Furthermore, SHERLOCK has been shown its capability of sensitive detection, discrimination and identification, genotyping and so on. Finally, the lateral flow (dipstick)-based test (“paper test”) cost down to $0.61 per reaction is much lower than ddPCR. As a CRISPRdx tool, SHERLOCK has been updated within several months [115]. Now SHERLOCK v2 holds four significant advantages over the first version (Fig. 3f): (1) more sensitive (down to zeptomolar level); (2) more convenient with portable lateral flow strips; (3) multiplex detection using Cas12a, Cas13 and Csm6 together; (4) turned into a quantitative detection approach. More recently, non-specific ssDNase cleavage (collateral cleavage like Cas13) of Cas12a was discovered and applied to establish a DNA detection system with attomolar sensitivity called “DETECTR” [116]. In the future, different Cas enzyme systems could be combined to simultaneously function in a single cell. Multicolor imaging will be realized via catalytically inactive Cas effector orthologs and many others labeled with different fluorescence [117].

### Applications of dCas13a

So far, the feasibility of CRISPR/dCas13a in engineering interference against RNA viruses has been proven [118]. Other applications of dCas13a can include: (1) specific RNA imaging, visualization, tracking of transcripts in living cells when fused with a fluorescent protein [119]; (2) sequestration, trafficking, editing of a specific RNA [120]; (3) modulation at transcriptional and translational levels via fusion with a transcriptional repressor or activator; (4) identification of specific RNA-associated proteins; (5) specific RNA editing like reported Cas13b-based RNA editing CRISPR system (REPAIR) system [120].

### CRISPR/Cas13b

Cas13b (formerly c2c6) is another RNA-guided RNA-targeting system with collateral effect. With Cas13b alone or with Csx27, the system might be more specific than Cas13a because RNA targeting is dependent on a double-sided PFS with a D (A, U, or G) at 5′ end and NAN/NNA at 3′end (Fig. 2). Distinguished from Cas13a, Cas13b are CRISPR-associated RNA-guided RNase with two crRNA variants [121]. Cas13b systems lack cas1 and cas2 and its activity could be repressed by Csx27 protein and enhanced by Csx28 [121, 122]. Although the mechanisms on how the two accessory proteins co-function have not yet been revealed, scientists have already tested the capability of Cas13 in nucleic acid detection [116] and RNA editing [120]. The first accurate REPAIR system exploits PspCas13b for gene knockdown along with ADAR2 deaminase activity (Fig. 3h), by using its highest level of interference (average knockdown 62.9%) among three Cas13 enzymes (Cas13a, b and c) [120]. This system can change adenosine to inosine (a base that functionally mimics guanosine in many cellular reactions) for replacement of a reporter gene, endogenous transcripts and known pathogenic mutations. Repairing of G to A pathogenic mutations or making loss-of-function of RNA via introduction of terminating codons can be achieved in two steps: targeting A specified by mismatching C on gRNA and converting target A to I by using dCas13b-ADAR2_DD_ (E488Q/T375G) fusions, which has been updated by REPAIRv2 with more than 919-fold higher specificity [120]. One possible future direction is to use dCas13b fused with APOBEC1 for the cytosine targeting and editing. Hence, REPAIR technology may become more and more significant for gene therapy and other biotechnology applications.

### Potential limitations of CRISPR/Cas13

Several issues that may inhibit the development of the CRISPR/Cas13 system should be cocerned: (1) RNA editing of small RNA target (< 22 nt) is limited, because crRNA needs to be long enough for recognition [112]; (2) possible off-target activity of CRISPR/Cas13 is an issue. Cas13 could be engineered to enhance specific targeting [123]; (3) effective ssRNA cleavage could be toxic in eukaryotic cells [112, 124].

## Conclusions

**Fig. 4:**
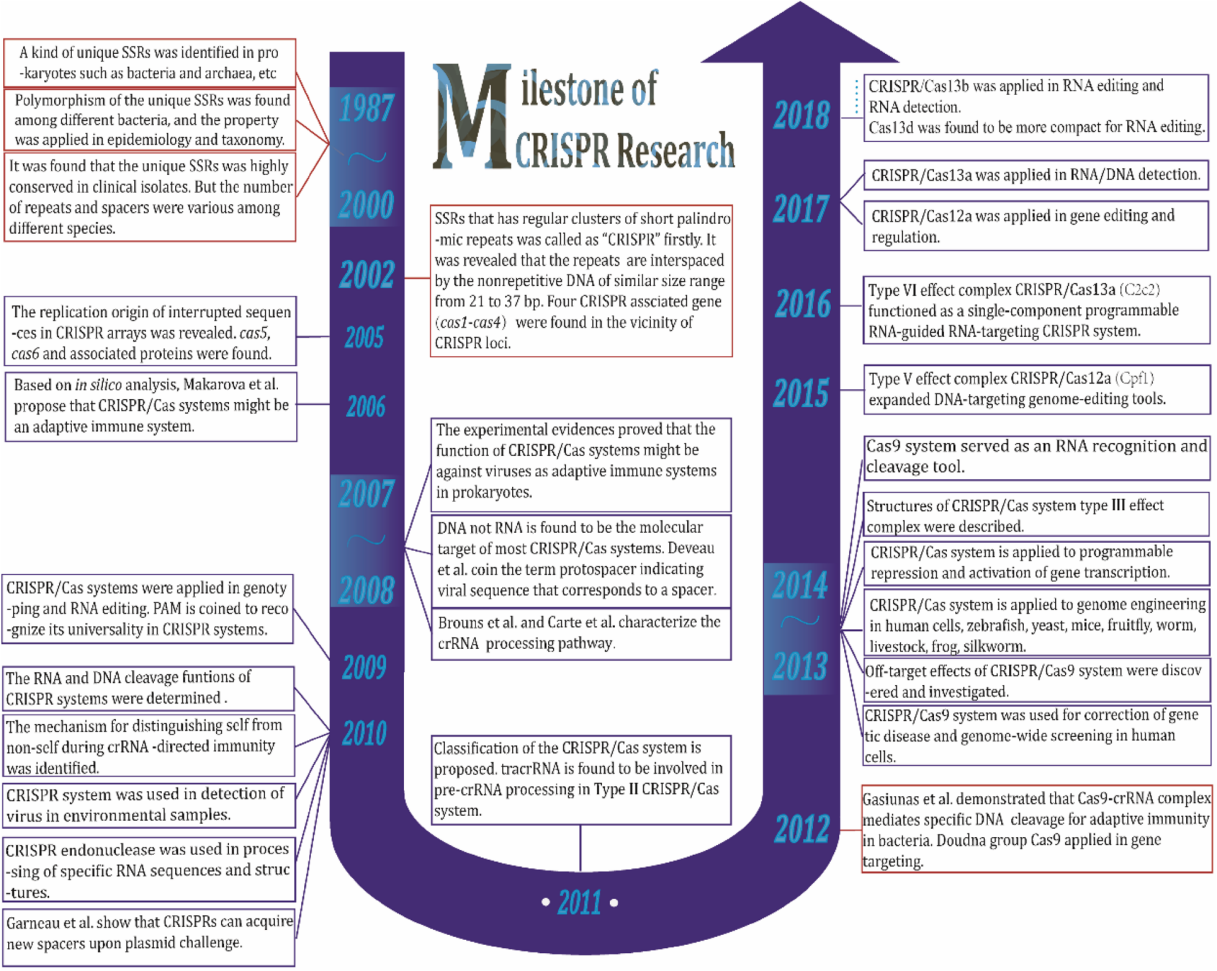
The milestone of CRISPR/Cas research development and achievement

Discovery of the well-known CRISPR/Cas9 system is a historical leap in modern biology (Fig. 4), especially for genome editing. It has been revealed that Cas9-mediated genomic cleavage induces cellular toxicity within the cells [125, 126]. Hence, minimizing DSB in genome-editing might be a better modality. Recently, base editing technologies, e.g. adenine base editors (ABEs), have emerged. Researchers exploited Cas9 nickases (Cas9n) for DNA targeting without DSB [127, 128]. In this way, base editing will introduce less off-target and cause fewer indels (typically ≤ 0.1%) than regular CRISPR/Cas-mediated gene editing. The fusion of a Cas9n and a base-converting enzyme is programmed with a guide RNA like aforementioned RNA-editing REPAIR systems [120]. However, base editing could realize only four transition mutations (G ↔ A, C ↔ T) now. So, improvement of its performance in different cells and more transition modes are expected. In another hand, immunogenicity of Cas proteins may be a drag on CRISPR therapeutics. Porteus et al. [129] reported that there were pre-existing humoral and cell-mediated adaptive immune responses to Cas9 (SaCas9 and SpCas9) in humans, triggering a heated debate on CRISPR therapeutics, although their data was obtained from in vitro experiments. Considering the required safety in clinical trials, researchers will pay more attention on the immunogenicity of Cas proteins to eliminate undesired immunity safety issues in preclinical stages.

In this review, we advanced the understanding of staggered Cas9 cleavage pattern. More new cleavage mechanisms will be discovered with better understandings of some more related systems (Table 1). New Cas enzyme systems also have great potential for targeted gene therapy of human and animal diseases, targeted mutagenesis in plants for crop improvement, or complex transcriptome patterns reprogramming of cells [117]. Emerging Cas12a and Cas13a systems have an immediate impact on biotechnology development. The former has improved the fat content of soybeans [130] and the latter was used to treat several RNA viruses [112]. Unlike Cas12a, Cas13b systems with a double-sided PFS and a regulation module are more likely used for applications with enhanced targeting specificity. Remarkably, the newly-discovered Cas13d, the smallest effector to date (~ 930aa) [131], is expected to exhibit better performance than others for transcriptome editing [132]. Although Cas13c (~ 1120aa) is still in the early stage of functional characterization, its potential should not be underestimated because of its two HEPN domains [131]. Further detailed structural studies and functional validation of Cas13c in cells will be vital for defining their mechanistic differences and functional efficiency. Now we can realize DNA editing using Cas9 and Cas12a systems in GC-rich and AT-rich regions, respectively. Considering of cytotoxicity, Cas12a could be used instead of Cas9 in some species like *C. glutamicum*. We can employ RCas9 or Cas13 systems for RNA editing as well. The applications beyond genome editing of CRISPR/Cas and dead-Cas proteins are reviewed further. Cas9-based tools performed well in NGS normalization, molecular diagnosis, transcriptional regulation, living cells imaging and localizing, etc. Detection or diagnosis using Cas12a and Cas13 systems showed high specificity because of the collateral effect. This advantage could be of great benefit for multicolor nucleic acid imaging, engineering interference and regulation against RNA, identification of specific DNA/RNA-associated proteins and so on. Future discovery and characterization of divergent CRISPR systems will benefit further expansion of CRISPR-based tools for and beyond genome editing applications.

**Table 1:** Current characterization of Type II, V and VI effectors

Effector^a^	Cas9	Cas12a	Cas13a	Cas13b	Cas13d
Subtype	II-A	V-A	VI-A	VI-B	VI-D
Target	dsDNA	dsDNA	ssRNA	ssRNA	ssRNA
Nuclease domain^b^	RuvC-NTS, HNH-TS	RuvC-NTS, TS Nuc	2 HEPN	2 HEPN	2 HEPN
Pre-crRNA processing	RNase III	WED III	Helical 1 or HEPN2	?	?
tracrRNA	Yes	No	No	No	No
Cut nature	Staggered, 5′ 1- or 3-nt overhangs	Staggered, 5 nt overhangs collateral activity-ssDNA	Collateral activity-ssRNA	Collateral activity-ssRNA	Collateral activity-ssRNA
PAM/PFS^c^	3′G-rich PAM	5′T-rich PAM	3′ PFS: H	5′PFS: D;3′ PFS:NAN or NNA	No?
Median size	1228 aa	1268 aa	1228 aa	1128 aa	928 aa

^a^ Characterized and validated types are included

^b^ TS and NTS indicate domain that cleaves target strand (TS) or non-target strand (NTS)

^c^ D: A, G, or U; H: A, C, or U; N: A, C, G, or T (or U)

## Abbreviations

CRISPRs: clustered regularly interspaced palindromic repeats
SRSRs: short regularly spaced repeats
DRs: direct repeats
LS: leader sequence
REC: recognition lobe
NUC: nuclease lobe
sgRNA: single guide RNA
REPAIR: RNA editing for programmable A to I replacement
HDR: homology-directed repair
NHEJ: non-homologous end joining
ZFN: zinc-finger nuclease
TALENs: transcription activator like effectors
DSB: double strand break
PAM: protospacer adjacent motif
Cas9: CRISPR associated protein 9
dCas9: dead or deactivated Cas9
RCas9: RNA-targeting Cas9
STRs: short tandem repeats
crRNA: CRISPR RNA
SHERLOCK: specific high sensitivity enzymatic reporter unlocking
EGFR: epidermal growth factor receptor
HEPN: higher eukaryotes and prokaryotes nucleotide-binding
PFS: protospacer-flanking site
RPA: recombinase polymerase amplification
CRISPRdx: CRISPR-based diagnostics
DETECTR: DNA endonuclease targeted CRISPR trans reporter
TS: target DNA strand
NTS: non-target DNA strand
